# Characterization of the complex formed between a potent neutralizing ovine-derived polyclonal anti-TNFα Fab fragment and human TNFα

**DOI:** 10.1042/BSR20130044

**Published:** 2013-08-23

**Authors:** W. Mark Abbott, Melanie Snow, Sonia Eckersley, Jonathan Renshaw, Gareth Davies, Richard A. Norman, Peter Ceuppens, Jerry Slootstra, Joris J. Benschop, Yoshitomo Hamuro, Jessica E. Lee, Peter Newham

**Affiliations:** *AstraZeneca, Alderley Park, Macclesfield, Cheshire SK10 4TG, U.K.; †Pepscan Presto, Zuidersluisweg 2, 8243 RC Lelystad, The Netherlands; ‡ExSAR, 11 Deer Park Drive, Suite 103, Monmouth Junction, NJ 08852, U.S.A.

**Keywords:** biotherapeutic, epitope, Fab, polyclonal, sepsis, TNFα, CLIPS, chemically linked peptides on scaffolds, DMEM, Dulbecco’s modified Eagle medium, HDX-MS, hydrogen/deuterium exchange MS, hTNFα, human TNFα, mAb, monoclonal antibody, mTNFα, mouse TNFα, rTNFα, recombinant TNFα, SEC, size exclusion chromatography, SPR, surface plasmon resonance, TNFα, tumour necrosis factor α, TNF-R, TNF receptor

## Abstract

TNFα (tumour necrosis factor α) is an early mediator in the systemic inflammatory response to infection and is therefore a therapeutic target in sepsis. AZD9773 is an ovine-derived, polyclonal anti-TNFα Fab fragment derived from a pool of serum and currently being developed as a treatment for severe sepsis and septic shock. In the present study, we show that although AZD9773 has a modest affinity for TNFα in a binding assay, the K*_i_* in a cell-based assay is approximately four orders of magnitude lower. We show using SEC (size exclusion chromatography) that the maximum size of the complex between AZD9773 and TNFα is consistent with approximately 12 Fabs binding to one TNFα trimer. A number of approaches were taken to map the epitopes recognized by AZD9773. These revealed that a number of different regions on TNFα are involved in binding to the polyclonal Fab. The data suggest that there are probably three epitopes per monomer that are responsible for most of the inhibition by AZD9773 and that all three can be occupied at the same time in the complex. We conclude that AZD9773 is clearly demonstrated to bind to multiple epitopes on TNFα and suggest that the polyclonal nature may account, at least in part, for the very high potency observed in cell-based assays.

## INTRODUCTION

TNFα (tumour necrosis factor α) is a potent pro-inflammatory cytokine that is the first to be released in response to infection and can trigger the complete spectrum of responses characteristic of endotoxic shock [1–3]. The key role that TNFα plays in the inflammatory response has made it a very attractive target in inflammatory diseases, and biologic agents are now widely used to suppress its action in rheumatoid arthritis, psoriasis and Crohn's disease [3–5]. However, despite clear evidence of a pivotal role in septic shock, anti-TNFα agents have had limited success in treating patients with these symptoms [6,7].

TNFα is a non-covalent homotrimer with a subunit molecular mass of 17 kDa, first isolated in 1985 from tissue culture supernatant of a phorbol ester stimulated cell line [8]. It is initially synthesized as a membrane-bound form that is then cleaved to release the soluble cytokine by TACE (TNFα-converting enzyme) [9]. The crystal structures of both hTNFα (human TNFα) and mTNFα (mouse TNFα) molecules have been determined [10,11]. Each monomer consists of a β-pleated sheet sandwich, which forms the trimer. TNFα exerts its effects through two distinct receptors: TNF-R1 and TNF-R2 [12]. Several different approaches have been used to study the interaction of TNFα with its receptor. Banner et al. published the structure of LTA (lymphotoxin α), which shares 33% sequence identity with TNFα in complex with TNF-R1 [13]. This study showed that the receptor binds at the interface of the subunits with three receptor molecules binding to one trimer, with two main sites of interaction on the ligand. No structure exists for the TNFα:receptor complex that definitively identifies the area of receptor interaction. Site-directed mutagenesis studies have, however, indicated that residues essential for receptor binding are located at the interface of the subunits [14–16]. Two early studies with polyclonal antibody or mAb (monoclonal antibody) suggested that the N-terminus and the residues 25, 27 and 144 might be involved in, or be in close proximity to, receptor recognition sites [17,18].

Clinically used anti-TNFα agents have been characterized for their interaction with TNFα in a number of different studies (US patent no. 6,284,471 [19,20]). These studies have revealed a number of different epitopes, affinities and sizes of the complexes formed. In addition, a number of other studies have identified up to six different epitopes on recombinant hTNFα using mAb and polyclonal antibody, with three of these appearing to be neutralizing epitopes [18,21–24]. The aim of this study was to investigate at a detailed molecular level how AZD9773 interacts with TNFα, in particular with respect to affinity and potency, the size of the complex, different epitopes and to understand how these compare with some of the characterized anti-TNFα biological agents used in the clinic.

## EXPERIMENTAL

### Preparation of AZD9773

AZD9773 is an ovine-derived, polyclonal Fab fragment obtained from sheep immunized with hTNFα. Merino wether sheep were immunized with recombinant human TNFα and maintained at the BTG facility (Martindale Holdings Pty Ltd, Mintaro, South Australia, Australia). Blood was recovered at approximately 4-weekly intervals at processing facilities at Turretfield Research Centre (Rosedale, South Australia, Australia) according to strict state and national ethical guidelines for animal welfare. The animals were not terminally bled. Sheep antibodies are very similar in structure to human antibodies [25]. Isolated papain-generated Fabs are >95% pure (see [26] for an overview of polyclonal-derived biotherapeutics). In all cases, the protein was initially reconstituted in PBS at 10 mg/ml.

### Preparation of TNFα proteins

Mature hTNFα (P01375) and mTNFα (P06804) were obtained from R&D Systems, Boehringer or were expressed in *Escherichia coli* and purified as described below.

Soluble mTNFα mutant DNAs (Table 1) were synthesized and cloned by GeneArt Life Technologies. The proteins were expressed in *E. coli* (BL21-Gold; DE3) at 18°C and then a lysate supernatant purified by a three-step process using anion exchange (Q Sepharose FF GE), hydrophobic interaction (phenyl Sepharose HP GE) and gel filtration (Superdex 200 GE) chromatography.

**Table 1:** Amino acid changes for TNFα mutants 1–20 Mutants 14–17 and 18–20 were changed progressively from mTNFα to hTNFα. The underlined residues are the additional residues in each mutant, where 13–17 are one set of mutants and 13 followed by 18–20 are a second set.

Mutant	Amino acid changes
1	[Q6R, N7T, S8P]mTNF-α(1–156)
2	[Q6R, N7T, S8P, A52S, D53E]mTNF-α(1–156)
3	[S30N, Q31R]mTNF-α(1–156)
4	[D71S, Y72H, ins 71_T_72]mTNF-α(1–156)
5	[P101Q, K102R, D103E, L110A]mTNF-α(1–156)
6	D71S, Y72H, ins 71_T_72, P101Q, K102R,D103E,L110A]mTNF-α(1–156)
7	[E88T]mTNF-α(1–156)
8	[L137R, K139D]mTNF-α(1–156)
9	[H20P, V22A, E24G, E27Q]mTNF-α(1–156)
10	[H20P, V22A, E24G, E27Q, S30N, Q31R, L137R, K139D]mTNF-α(1–156)
11	[Q130R]mTNF-α(1–156)
12	[D42E, K44R, F82I, I84V, E88T, Q130R]mTNF-α(1–156)
13	[M41V, V58I, V79I, V96I, V135I, V153I]mTNF-α(1–156)
14	[H20P, V22A, E24G, E27Q, M41V, V58I, V79I, V96I, V135I, V153I]mTNF-α(1–156)
15	[H20P, V22A, E24G, E27Q, S30N, Q31R, M41V, V58I, V79I, V96I, V135I, L137R, K139D, V153I]mTNF-α(1–156)
16	[L1V, Q6R, N7T, S8P, H20P, V22A, E24G, E27Q, S30N, Q31R, M41V, V58I, V79I, V96I, V135I, L137R, K139D, V153I]mTNF-α(1–156)
17	[L1V, Q6R, N7T, S8P, H20P, V22A, E24G, E27Q, S30N, Q31R, M41V, D42E, K44R, A52S, D53E, V58I, V79I, F82I, I84V, E88T, V96I, Q130R, V135I, L137R, K139D, V153I]mTNF-α(1–156)
18	[L1V, Q6R, N7T, S8P, H20P, V22A, E24G, E27Q, S30N, Q31R, M41V, V58I, V79I, V96I, V135I, V153I]mTNF-α(1–156)
19	[L1V, Q6R, N7T, S8P, H20P, V22A, E24G, E27Q, S30N, Q31R, M41V, D42E, K44R, V58I, V79I, V96I, P101Q, K102R, D103E, L110A, V135I, L137R, K139D, V153I]mTNF-α(1–156)
20	[L1V, Q6R, N7T, S8P, H20P, V22A, E24G, E27Q, S30N, Q31R, M41V, D42E, K44R, V58I, D71S, Y72H, ins 71_T_72, V79I, V96I, P101Q, K102R, D103E, L110A, V135I, L137R, K139D, V153I]mTNF-α(1–156)

### Analysis of AZD9773 binding to TNFα

A detailed kinetic analysis of the binding of AZD9773 to hTNFα and mTNFα was performed using SPR (surface plasmon resonance) on a Biacore 3000 instrument. AZD9773 Fab fragments were immobilized using amine-coupling chemistry to achieve a ligand immobilization level of 800RU. A 12-point concentration series of TNFα samples was prepared using three-fold serial dilutions from 30 μM. TNFα samples were injected across the sensor surface for 360-s association times, with 600-s dissociation. The lowest concentration of TNF (0.17 nM) was injected first, followed by the samples of increasing TNFα concentration. Each TNFα injection was interspersed with a single 20-s wash injection of 10 mM glycine, pH3.0. The affinity was determined by two methods: equilibrium binding once steady-state binding had been reached, and an analysis of the *k*_a_ (association rate constant) and *k*_d_ (dissociation rate constant).

Mutant TNFα proteins were analysed for their binding to AZD9773 using SPR on a Biacore 3000 or 1000 instrument. The TNFα proteins were immobilized onto a CM5 sensor chip to give approximately 1000RU. AZD9773 was injected over this surface at a concentration of 100 μg/ml in 20 mM sodium phosphate (pH7.5), 150 mM sodium chloride and 0.005% P20 surfactant (Biacore) at a flow rate of 30 μl/min for 3 min. The association value in RU was recorded at the end of the 3-min injection. hTNFα and mTNFα were used as positive and negative controls, respectively.

### Analysis of efficacy of AZD9773 in TNFα-mediated cytotoxicity assay

L929 (mouse fibroblast) cells were cultured in DMEM (Dulbecco's modified Eagle medium), 5% (v/v) FBS and 2 mM GlutaMAX at 37°C in 5% (v/v) CO_2_. Cells were plated in DMEM containing 1% FBS and 2 mM GlutaMAX into 96-well tissue culture plates with 1.25×10^4^ cells in 50 μl being dispensed into each well and the cells incubated for 20 h at 37°C. TNFα proteins and AZD9773 were diluted in DMEM and 1% FBS. Indicated concentrations of TNFα and Fab were premixed and 100 μl added to each well after the 50 μl of plating media had been removed. In order to determine the EC_50_ dose for the different TNFα molecules, a dilution series of 0.02 pg/ml to 40 ng/ml was used. Cells were then incubated for a further 20 h before viability was determined by adding 20 μl of CellTitre 96AQ One solution reagent (Pierce). The plate was then incubated for a further 4 h at 37°C, 5% CO_2_. Absorbance values were recorded after a 5-s shake using a microtitre plate reader at a wavelength of 490 nm.

Cell survival data were analysed by fitting three four-parameter logistic curves to the three separate treatments (TNF alone, AZD9773 41 ng/ml+TNF and AZD9773 1200 ng/ml+TNFα) versus the TNF concentration on each plate. The curves were fitted with common asymptotes on each plate (top, cell survival and bottom, cell death) for the three curves and a common slope. The EC_50_ was allowed to vary. For each plate, the shift in the AZD9773 curves relative to TNF alone was calculated and quoted as a fold change.

Data were summarized by calculating a geometric average of the fold changes for each mutant and the human and mouse controls and obtaining a pooled estimate of variability across all of the mutants and controls. There was evidence that this pooled variability differed depending on the dose of AZD9773, so separate calculations of pooled variability were produced for each dose.

### Analysis of AZD9773:hTNFα complex using SEC (size exclusion chromatography)

The hTNFα:AZD9773 complex was investigated using SEC. A Superdex 200 PC3.2/30 column (GE Healthcare) was equilibrated into PBS+azide on an Ettan system (GE Healthcare) at a flow rate of 50 μl/min. hTNFα (Boehringer Ingelheim GmbH) and AZD9773 were incubated at a molar ratio of 20, 40, 80 and 200:1 (AZD9773:trimeric TNFα), keeping either AZD9773 or hTNFα constant, respectively. Incubations were carried out at room temperature (20°C) overnight and the complexes were subjected to SEC analysis in 50 μl volumes. TNFα (8.98 mg/ml; ~172.5 μM) and AZD9773 (11.9 mg/ml; ~238 μM) were also analysed uncomplexed in 10 μl volumes. Chromatograms were obtained by monitoring the absorbance at 280 nM. A set of molecular standard markers (Sigma MWGF1000-1KT) ranging from 29 to 669 kDa was used to calibrate the column. The apparent molecular mass of samples was estimated by fitting the retention/elution volume to the calibration curve obtained from the molecular mass standards.

### Epitope mapping using linear and constrained peptide libraries

A total of 2500 different peptides were generated using proprietary CLIPS (chemically linked peptides on scaffolds) technology (Pepscan), peptide arrays that reconstruct conformational and discontinuous epitopes of hTNFα [27]. These were synthesized and screened as first described by Slootstra et al. in 1996 [28]. The peptides included all overlapping linear 15- and 30-mer peptides, all overlapping single-looped 15-mer peptides, and matrix scans that reconstruct discontinuous epitopes.

The libraries were screened for binding by incubation with AZD9773 at three different concentrations: 1, 10 and 100 μg/ml.

### Epitope mapping using HDX-MS (hydrogen/deuterium exchange MS)

The general H/D exchange epitope mapping procedure has been described previously [29]. Briefly, exchanged protein samples were added to 2 M urea and 1 M TCEP [tris-(2-carboxyethyl)phosphine; pH3.0] and passed over an immobilized pepsin column at 0°C. The digested fragments were separated by a C18 column and mass spectrometric analyses were performed with a Thermo LTQ™ mass spectrometer (Thermo Fisher).

## RESULTS

### Generation of AZD9773

AZD9773 is >95% pure Fab, as observed by SDS/PAGE and analytical SEC. Approximately 10% of the product was able to bind hTNFα, as determined by affinity purification (results not shown). Although the product could be affinity purified on a TNFα column, this was not done as it has two disadvantages: firstly, it would have required very large quantities of TNFα, and secondly, the elution conditions would likely elute some TNFα as it is a non-covalent trimer.

### Characterization of AZD9773 binding to TNFα

**Figure 1:**
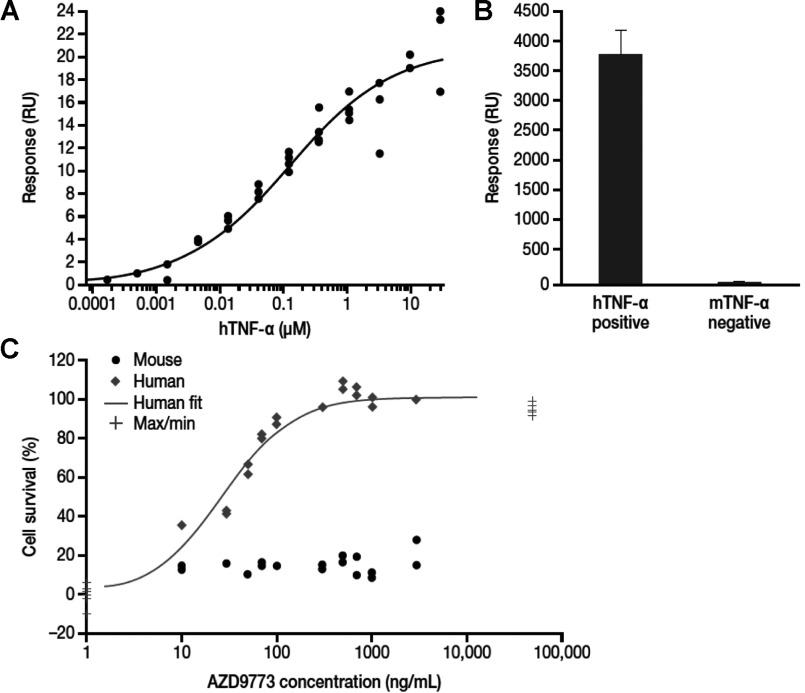
Analysis of binding affinity and efficacy of AZD9773 (**A**) The steady-state fit for hTNF-α binding to immobilized AZD9773 at the 360 s time point is shown. A full dose response was performed three times on two separate occasions. Fitting the data to a steady-state binding model enabled the *K*_d_ to be calculated as 124 nM (95% CI, 65–235 nM). (**B**) TNF-α was immobilized and AZD9773 binding determined by injection at 100 μg/ml for 3 min. The response was measured in RU and normalized to 1000 RU immobilized TNF-α. The measurements are means±S.D. *C*, the *K*_i_ for AZD9773 against hTNF-α was calculated using the Cheng-Prusoff equation: *K*_i_=IC_50_/(1+(A/ED_50_)), where IC_50_ is the concentration that produces 50% inhibition, ED_50_ is the dose that produces 50% of the maximum achievable effect and A is the concentration of hTNF-α. This revealed a *K*_i_ of 36 pM.

The binding of AZD9773 was analysed using two separate approaches for SPR. The affinity of AZD9773 was determined by immobilizing the Fab directly onto a sensor chip, injecting different concentrations of hTNFα, and then allowing the system to reach steady-state binding after 360 s. 800RU of Fab was immobilized to the sensor chip surface, which equates to approximately 80RU of anti-TNFα Fab. Higher levels of Fab immobilization resulted in non-saturable binding suggestive of non-specific binding and thus were not used. When TNFα over a concentration range of up to 30 μM was injected over the surface, the apparent *K*_d_ (dissociation constant) for binding was 124 nM (Figure 1A). However the kinetic analysis was complex, with a Hill slope of 0.5 tentatively suggesting multiple phases in the binding interaction. The presence of lower-affinity Fabs may bias the *K*_d_ according to the concentration range measured. Consequently, different values for *K*_d_ were obtained, depending on the concentration range of TNFα employed (for TNFα ≤1 μM, *K*_d_ was 17 nM [95% CI, 14–20], whereas testing to a maximal concentration of 30 μM gave a *K*_d_ of 62 nM) (Figure 1A). It seems likely that this reflects the polyclonal nature of AZD9773 with Fabs possessing a range of affinities.

The specificity of AZD9773 was determined using SPR, but in this case TNFα was immobilized and then AZD9773 injected over the surface. This showed that while AZD9773 bound strongly to hTNFα, it did not bind above background levels to mTNFα (Figure 1B). AZD9773 did not bind rat or porcine rTNFα (recombinant TNFα) but did bind canine and primate rTNFα (results not shown).

### Characterization of potency of AZD9773

The ability of AZD9773 to inhibit TNFα-mediated cellular toxicity was evaluated using L929 cells. The *K*_i_ (inhibition constant) for AZD9773 versus hTNFα was determined to be 36pM (Figure 1C). As expected, AZD9773 did not inhibit murine rTNFα biological activity. As AZD9773 only contains about 10% of Fabs that bind hTNFα, the real *K*_i_ may be as low as about 4 pM. Thus, the potency in this assay is much greater than the mean hTNFα-binding affinity.

### Determination of size of AZD9773/TNFα complex

**Figure 2:**
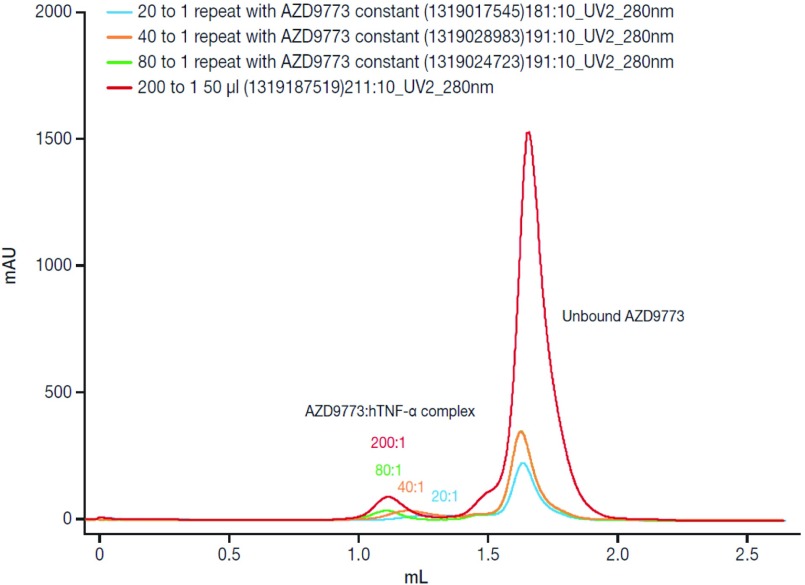
Analysis of the size of the AZD9773/TNF-α complex using SEC AZD9773 and hTNF-α were mixed at the ratios shown for 16 h at room temperature and then injected over a Superdex 200 column equilibrated in PBS. The column was equilibrated with molecular mass standards of between 29 and 669 kDa.

Analysis showed that at AZD9773:hTNFα ratios >40:1, all TNFα was complexed by the Fab and that the size of the complex increased with increasing ratio to reach a maximum of about 650 kDa at 80:1 (Figure 2). This suggests that approximately 12 Fabs bind one TNFα trimer. It is difficult to predict the exact value owing to the limitations of SEC as the shape of the complex is unknown and an elongated structure would appear to have an artificially high molecular mass. However, the polyclonal hTNF binding nature of AZD9773 is apparent from this data.

### Epitope mapping of AZD9773

We then attempted to define more precisely the location of binding sites on TNFα for the Fabs through three different approaches.

### Peptide-based methods

**Figure 3:**
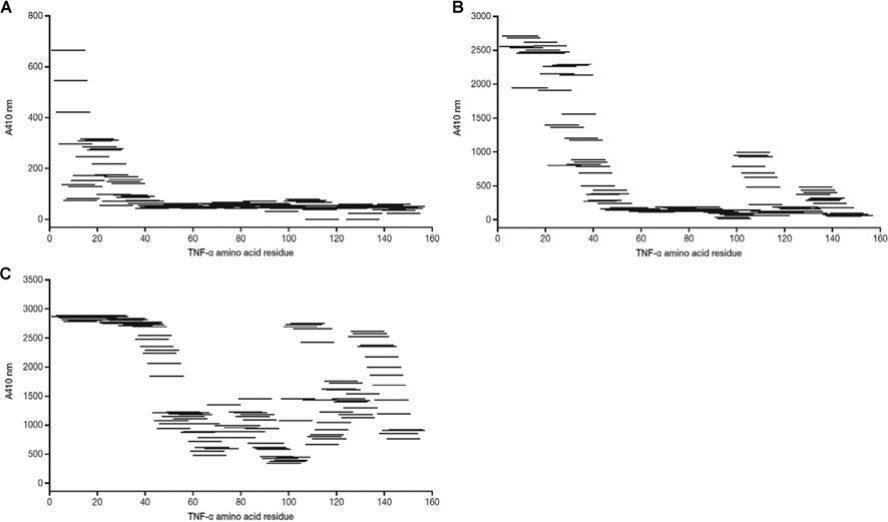
Two-dimensional alignment of single-loop 15-mer peptides according to TNF-α sequence (*x*-axis) and observed Pepscan ELISA value (*y*-axis) (**A**) 1 μg/ml sample concentration. (**B**) 10 μg/ml sample concentration. (**C**), 100 μg/ml sample concentration.

In the first approach, a number of different linear and constrained peptide libraries were screened. The constrained libraries were built to try and mimic discontinuous epitopes. The peptides were screened for binding to AZD9773 by ELISA. The data for single-looped peptides are shown in Figure 3. Data from the linear and other structurally constrained libraries identified the same regions (not shown). The analysis identified five regions on the TNFα molecule that were specifically bound by AZD9773. These are: _1_VRSSSR_6_, _17_VANPQAEG_24_ and _29_LNRR_32_ (observed at low and high concentrations), and _105_TPEGAEA_111_ and _135_EINRP_139_ (observed at higher concentrations only). When sample concentrations were increased even further, no additional binding regions were identified. The identified five binding regions are all located on the outside of the TNFα trimer, and at least four of the regions are in close proximity to the proposed binding sites of the TNFα receptor.

### HDX-MS

In the second approach, the epitope of TNFα against AZD9773 was identified by HDX-MS [29,30]. Two different types of HDX-MS experiments of TNFα were performed: on-exchange without AZD9773, and on/off-exchange with and without immobilized AZD9773.

**Figure 4:**
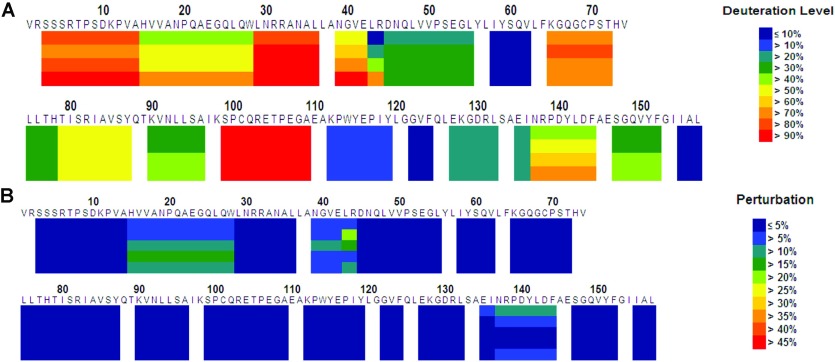
HDX-MS for epitope mapping of AZD9773 (**A**) On-exchange results of hTNF-α at 0°C, pH7.0. Each block represents peptide analysed and contains four time points: 150, 500, 1500 and 5000 s. The deuteration level at each time point for each peptide is colour coded, as shown on the right. (**B**) Each block represents a segment monitored and contains five time points: 150 and 500 s at 3°C, pH7.0; 150, 500 and 1500 s at 23°C, pH7.0 from top to bottom. Dark blue indicates no difference between the two on/off-exchange experiments. Other colours indicate more deuterium atoms after on-solution/off-column exchange than after on-column/off-column exchange, as shown on the right.

The on-exchange results of TNFα (Figure 4A) unveil the dynamic properties of TNFα in solution. TNFα is a moderately dynamic protein and its backbone amide hydrogen atoms exchange over a wide time range. Some regions of the protein, such as residues 99–109, were completely deuterated within the shortest time point employed (150 s at 0°C, pH7.0), while other regions, such as residues 58–62, 122–124 and 155–157, were not deuterated at all even after the longest time point (5000 s at 0°C, pH7.0). The on-exchange results suggest that a wide time window needs to be surveyed during on/off-exchange experiments for epitope mapping.

The on/off-exchange results of TNFα with and without immobilized AZD9773 (Figure 4B) indicate those regions of TNFα likely to be the HDX-MS-identified epitope. The backbone amide hydrogen atoms in the epitope should exchange more slowly when the antigen binds to its antibody. Therefore the backbone amides in the epitope should carry deuterium atoms after on-exchange in the ligand-free state (in solution) and after off-exchange in the antigen-bound state (in the column). To eliminate background noise, control experiments were also performed (on-column/off-column). The segments that carried significantly more deuterium atoms after on-solution/off-column than after on-column/off-column are the HDX-MS-identified epitopes. Two segments, residues 15–29 and 43–44, had significantly more deuterium atoms after on-solution/off-column experiments (>10% on average) than after on-column/off-column experiments. Two other segments, residues 39–43 and 137–144, had marginally more deuterium atoms (5–10%). All four segments were spatially close to each other.

### Mutagenesis of hTNFα sequence

The third approach taken to map the epitope(s) was to make a series of TNFα mutants (*n*=20) (Table 1). These mutants sought to explore the reasons for the difference in binding seen between hTNFα and mTNFα by using both a primary amino acid sequence alignment and superposed structures of hTNFα (PDB code 1TNF) and mTNFα (PDB code 2TNF) that highlighted ten areas on the surface of the protein that were in a proximity limit of about 20 Å (1 Å=0.1 nm) to each other. From this information, three approaches were used to design the mutants. A first set of 12 were made with essentially one region at a time changed from the mouse sequence to the human equivalent. Mutants 13–17 then progressively changed mTNFα to hTNFα in an additive manner. Mutant 13 contained six amino acid changes that were thought to be in the core of TNFα. The third set (mutants 18–20) started with mutant 13 and then progressively added changes based on the strength of evidence from the peptides, HDX-MS and the data from mutants 1–12.

All 20 mutants were successfully expressed and purified. They migrated as a single peak on a size exclusion column with a molecular mass consistent with being a trimer. The molecular mass as determined by electrospray mass spectrometry was in all cases as expected. The proteins were at least 80% pure when analysed by non-reducing SDS/PAGE and at least 90% pure with reducing SDS/PAGE.

**Figure 5:**
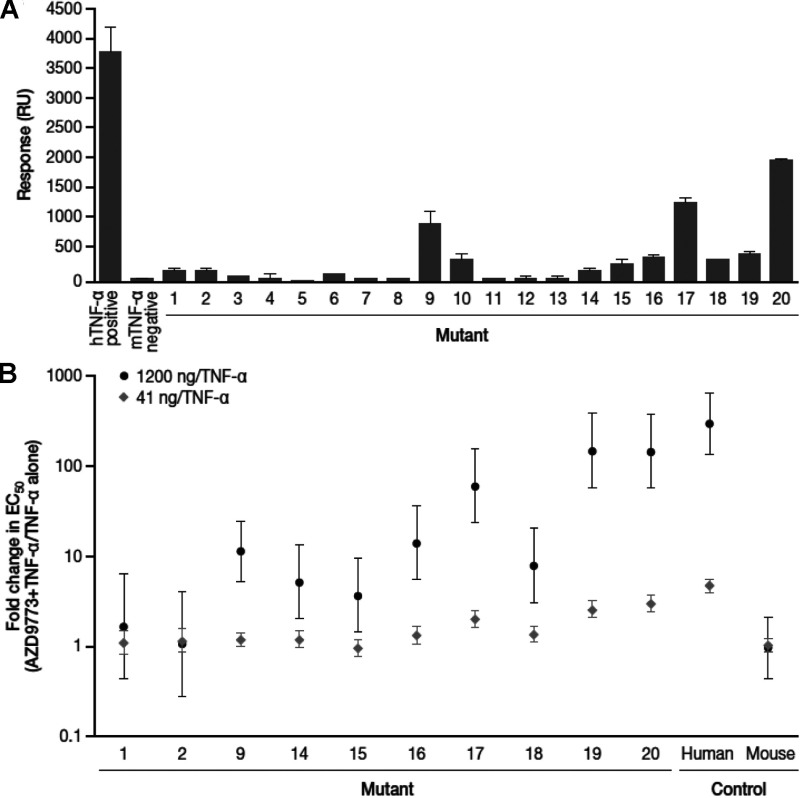
Analysis of mutant TNF-α proteins for their binding to AZD9773 and inhibition of their cytotoxicity by AZD9773 (**A**) Each mutant was immobilized on a separate channel of a CM5 sensor chip to give approximately 1000 RU. Binding of AZD9773 was determined by injecting 100 μg/ml for 3 min, after which the response was determined and corrected to 1000 RU. Values are means±S.D. of at least two experiments. (**B**) The cytotoxicity of each mutant was determined using a series of dilutions from 0.02 pg/ml to 40 ng/ml by itself and then in the presence of 41 ng/ml and 1200 ng/ml AZD9773. The graph shows fold changes in EC_50_ between AZD9773 (41 or 1200 ng/ml) and TNFα alone (error bars are 85% CIs).

The ability of these mutants to bind AZD9773 was evaluated using SPR (Figure 5A). In the first set of 12, mutants 9 and 10 bound most strongly; they had four common changes (H20P, V22A, E24G, E27Q) on a surface-exposed loop of TNFα and had been detected from the peptide and HDX-MS epitope mapping approaches. This loop would therefore appear to be a strong epitope. SEC showed a mass of about 230 kDa (results not shown) for the complex between mutant 9 and AZD9773, suggesting that just a single epitope was present. Weak binding was detected in mutants 1 and 2, which had three common changes near the N-terminus (Q6R, N7T, S8P). This region had been strongly identified from the peptide-based approaches but not HDX-MS. It therefore seems likely that this is an epitope. In the second set of mutants (13–17), binding increased as more amino acids were changed from mouse to the human equivalent, suggesting contributions from different parts of TNFα and therefore potentially multiple epitopes. However, it is noteworthy that even in mutant 17, 26 out of the 33 amino acid changes between mouse and hTNFα have been made, yet it still only binds approximately one-third as well as hTNFα, which suggests that the remaining seven residues (D71S, Y72H, Ins 71_T_72, P101Q, K102R, D103E, L110A) contribute very significantly to binding. Of the last set of three mutants, mutants 18 and 19 bound poorly, whereas mutant 20 bound well, although still only approximately 50% as well as hTNFα. As mutant 20 had 27 out of 33 changes, this suggested that the other six residues (A52S, D53E, F82I, I84V, E88T and Q130R) contributed significantly to binding. It is difficult to draw definitive conclusions from the data because there may be interactions between different residues that are changed in differing orders. However, taken altogether, the binding data suggest that there is an epitope around residues 20–27 and tentatively suggest epitopes around residues 71–73 and a patch around residues 52, 53, 82, 84, 88 and 130, along with smaller contributions from other regions such as the eight N-terminal amino acids.

Those mutants that bound AZD9773 were analysed for their ability to induce cytotoxicity in L929 cells (Supplementary Table S1, available at http://www.bioscirep.org/bsr/033/bsr033e060add.htm) and their susceptibility to inhibition by AZD9773 (Figure 5B). We have shown 85% CIs rather than 95% CIs and pairwise comparisons between mutants to indicate trends, as more amino acids are changed from mouse to their human equivalent. AZD9773 inhibited mutant 9, suggesting that the region between residues 20 and 27 is, or is at least part of, an inhibitory epitope. There was no clear inhibition of mutants 1 and 2. Although mutant 9 clearly binds AZD9773 and is inhibited in the L929 assay, there must be other parts of TNFα that contribute to the neutralizing activity of AZD9773 as the response is significantly less than with hTNFα.

The changes made between mutants 15 and 17 and then to hTNFα all contribute to an increased sensitivity to AZD9773, suggesting multiple inhibitory epitopes. With mutants 18–20, the largest increase in sensitivity occurred with the additional residues between 18 and 19. Mutant 19 is interesting as although it only binds AZD9773 quite weakly, it is almost as sensitive to inhibition as hTNFα. The complex between mutant 19 and AZD9773 was analysed by SEC and shown to have a molecular mass of about 500 kDa (results not shown), which suggests that there are likely to be three Fabs bound per monomer. Although these three potential epitopes only constitute just over 10% of the binding response, they would seem to be responsible for most of the AZD9773 efficacy. The inhibition data strongly indicate that the region between residues 20 and 27 is an inhibitory epitope and that there are other major contributions to inhibition from other epitopes, potentially encompassing the N-terminus and residues 42 and 44.

## DISCUSSION

The key findings from this study are that AZD9773 is a very potent functional inhibitor of TNFα, despite only having moderate binding affinity. AZD9773 is clearly shown to bind to multiple sites on hTNFα by virtue of the size of the complex in solution, as well as the presence of multiple epitopes as revealed by a combination of different approaches.

AZD9773 is a complex product as it not only represents a polyclonal antibody response to a protein antigen, it is also derived from pooled sheep serum. Thus, it might be expected to contain at least many tens of different antibodies against different epitopes. The product is a Fab fragment produced after papain cleavage, which results in different pharmacokinetic properties to a standard immunoglobulin therapeutic, notably a markedly shorter plasma half-life (cleared in hours rather than days) and more rapid tissue distribution, which may confer pharmacological advantages. In this study, we have sought to characterize the complex between AZD9773 and hTNFα in detail, with respect to the kinetics of binding, efficacy in a functional assay, molecular size and epitope determination for both neutralizing and non-neutralizing epitopes. In addition, as far as possible with a complex polyclonal product, we sought to compare the epitopes with those of commercial monoclonal antibodies.

Analysis of the binding data using SPR with the Fab immobilized showed a variable *K*_d_ depending on the concentration of TNFα injected over the surface. As AZD9773 is a polyclonal Fab, this is not surprising and the measured *K*_d_ values will almost certainly represent a wide range of affinities. In an assay based on TNFα-induced killing of L929 mouse fibroblasts, the active fraction of AZD9773 had a *K*_i_ of 4pM, which is approximately four orders of magnitude more potent than the average binding affinity. This might reflect the presence of a small fraction of very high-affinity antibodies, which are detected in an assay that uses TNFα at pM concentrations. Lewis and colleagues [31] recently suggested that the signalling competent tumour necrosis factor receptor 1 (TNFRI) molecule is a network induced by TNFα that results in a conformational change to an active state of the receptor. If this were the case, simple affinity of AZD9773 for the trimer may be very different to potency in cell-based assays, where binding to just one molecule in the network could result in a breakdown of that network and loss of signalling. Alternatively, it might be a consequence of a polyclonal antibody response being synergistic against different epitopes. As a first step toward clearly demonstrating and understanding the polyclonal nature of AZD9773, we characterized the size of the complex formed with hTNFα and defined the different epitopes involved in binding.

Analytical SEC clearly revealed that AZD9773 was capable of binding to multiple different sites on TNFα as the molecular size was consistent with approximately 12 Fabs bound per TNFα trimer. Although this is a very approximate size estimation of the complex because of the limitations of SEC, it is strongly suggestive that much of the TNFα surface will be covered with Fab molecules. The TNFα trimer has a surface area of approximately 19000 ^2^. Comparisons with published structures of antigens with Fabs very tentatively suggest that it could be possible to get 8–12 Fabs bound to the trimer, probably towards the lower end of this range. This conclusion was also suggested from experiments where we attempted without success to select out phage scFv molecules binding to TNFα from phage antibody libraries when using the complex for screening (results not shown).

**Figure 6:**
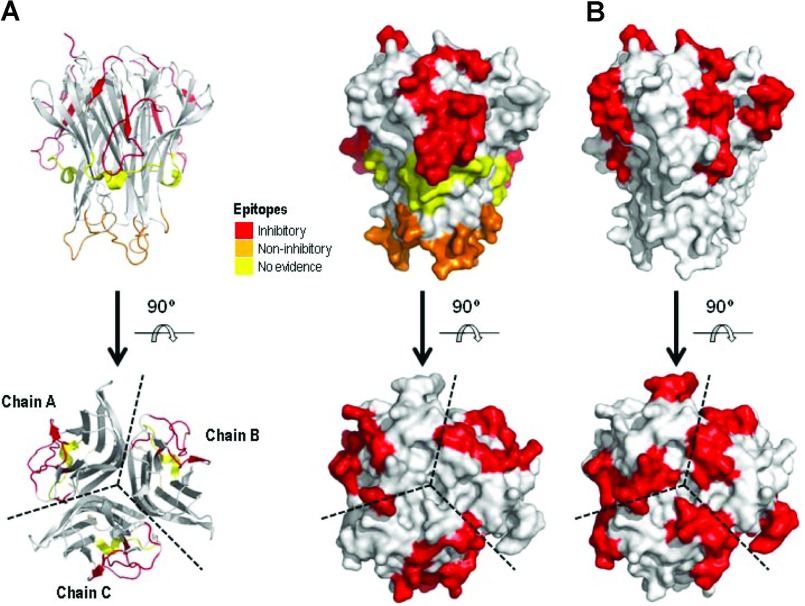
(A) hTNFα trimer:AZD9773 epitopes. (B) TNF receptor epitopes

Epitope mapping approaches further demonstrated the polyclonal nature of AZD9773 and have started to help us understand which epitopes do and do not contribute to efficacy. The most definitive way to map an epitope is to crystallize the antigen with the antibody, generally a Fab fragment [30,32,33]. However, this requires pure monoclonal antibody, which is in contrast to the polyclonal AZD9773 product. We therefore used three alternative approaches to derive a consensus on epitopes recognized by AZD9773: linear and constrained overlapping peptides, HDX-MS and mutagenesis to generate chimaeras of hTNF and mTNFα. An overall summary of the data is shown in Table 2 and the putative epitopes shown on TNFα structure in Figure 6 (an alignment of hTNFα, ovine and mTNFα sequences are shown in Supplementary Figure S1 at http://www.bioscirep.org/bsr/033/bsr033e060add.htm). It is clear from Figure 6 that there is significant overlap between the inhibitory epitopes and those parts of TNFα thought to interact with the TNF receptors. The region for which there is strongest evidence is between residues 15 and 32, which was identified by all three methods and encompasses an epitope required for biologic activity. Notably, this region contains a QXQ motif that Yone et al. in 1995 speculated to be part of a conformational inhibitory epitope together with F144, which interestingly is a tyrosine in the ovine sequence [18]. However, it is clear from the data on a mutant containing this region (mutant 9) that it only contributes to a small part of the binding activity and functional efficacy of AZD9773. In the first series of mutants (mutants 1–12), where one region at a time was changed from mouse to human, it was perhaps somewhat surprising that of the other mutant TNFα molecules, only those containing amino acid changes at the N-terminus bound to AZD9773 and then only weakly. This suggests that other epitopes are likely to result from combinations of changes that had not been explored. Therefore a second set of mutants was made where mTNFα was changed progressively to hTNFα. This second set of mutants clearly further demonstrated the polyclonal nature of AZD9773, as binding and sensitivity to inhibition increased with increasing number of changes. However, it was also clear that binding and inhibition did not necessarily change coincidentally with different mutants. This enabled a tentative definition of further inhibitory and non-inhibitory epitopes. Antibodies against the peptides derived from the N-terminus have been shown previously to be inhibitory [17]. Yone et al. in 1995 suggested that a goat polyclonal antibody reacts to residues 42–49, which encompasses this putative inhibitory epitope, although they did not attempt to determine if it was inhibitory [18].

**Table 2:** Classification of inhibitory and non-inhibitory epitopes Asterisks indicate the strength of evidence from a particular method supporting a region being an epitope.

Approximate region		HDX-MS	Peptides	Chimaeras	Class
1–8	VRSSSRTP	–	****	**	(Inhibitory)
15–32	HVVANPQAEGQLQWLNRR	****	****	****	Inhibitory
39–44	NGVELR	****	–	(**)	(Inhibitory)
71–73	STH	–	–	(****)	Non-inhibitory
102–111	QRETPEGAEA	–	*	(*)	Non-inhibitory
136–145	INRPDYLDFA	*	*	–	No evidence

The epitopes identified for two commercial anti-TNFα agents by Kim et al. in 2007 are clearly different to those identified for AZD9773 [19]. They identified a number of residues between 138 and 142 that, when mutated to glycine, bound infliximab and etanercept much more weakly. We did find some evidence from the peptide mapping and HDX-MS that this region was involved in binding. Owing to the nature of the mutants that were made, no definitive conclusions could be reached as to whether it is part of an inhibitory epitope for AZD9773.

Taken together, our data reveal the polyclonal nature of AZD9773 and suggest that this is a major determinant of the unexpectedly low apparent *K*_i_. Although the reason for this requires further work, phenomena similar to that reported by Marks in 2004 for multiple monoclonal antibodies to botulinum toxin may represent a plausible explanation [34]. He speculated that the increase in functional potency could result from conformational changes induced upon binding, resulting in increased affinity from other monoclonal antibody species and/or increasing the surface area of the toxin that interacts with the receptor. He showed that combining monoclonal antibody binding to non-overlapping peptides leads to a large increase in functional potency and an approximately 100-fold increase in binding affinity. In contrast, we used a polyclonal Fab, but it is conceivable that a similar type of phenomenon is being observed, especially when we consider that one of the AZD9773:hTNF binding regions we have identified (residues 15–32) contains the QXQ motif [18]. Thus, engagement of this region by Fab species in AZD9773 may enhance the affinity of other Fab species for their epitopes. In order to prove this, it would be necessary to clone out different antibodies from AZD9773 reactive to different epitopes using methods similar to those described by Cheung et al. in 2012 [35].

In summary, the data in this study show that AZD9773 is a very potent agent and also clearly reveal its polyclonal nature through a number of different approaches. We tentatively suggest that the polyclonal nature of AZD9773 may account in part for the very high potency.

## Online data

Supplementary data
