# Supplementary material for: Characterization of the complex formed between a potent neutralizing ovine-derived polyclonal anti-TNFα Fab fragment and human TNFα
Source: Biosci Rep. 2013 Aug 23;33(4):e00060. doi: 10.1042/BSR20130044 (PMC3755337; doi:10.1042/BSR20130044)
Supplement: Supplementary data [file bsr033e060add.pdf]

Biosci. Rep. (2013) / **33** / art:e00060 / doi 10.1042/BSR20130044

OPEN ACCESS

Original Paper

SUPPLEMENTARY DATA

Characterization of the complex formed between a potent neutralizing ovine-derived polyclonal anti-TNFα Fab fragment and human TNFα

W. Mark ABBOTT*[1], Melanie SNOW*, Sonia ECKERSLEY*, Jonathan RENSHAW*, Gareth DAVIES*, Richard A. NORMAN*, Peter CEUPPENS*, Jerry SLOOTSTRA†, Joris J. BENSCHOP†, Yoshitomo HAMURO‡, Jessica E. LEE‡ and Peter NEWHAM*

*AstraZeneca, Alderley Park, Macclesfield, Cheshire SK10 4TG, U.K., †Pepscan Presto, Zuidersluisweg 2, 8243 RC Lelystad, The Netherlands, and ‡ExSAR, 11 Deer Park Drive, Suite 103, Monmouth Junction, NJ 08852, U.S.A.

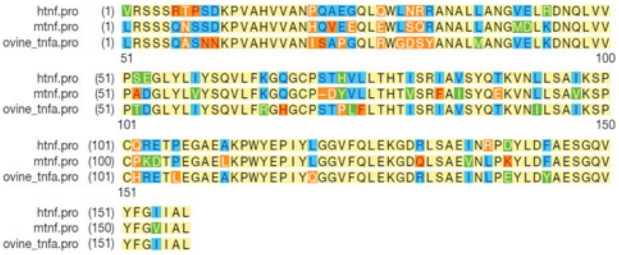

Figure S1 Alignment of hTNFα, mTNFα and ovine TNFα

Table S1 Potency of mutant TNFα proteins
CIR, confidence interval ratio.

				95 % CIs		
		GeoMean EC_{50}	*n*	Lower	Upper	CIR
Mutant	1	17	1	3	108	6.4
	2	36	1	6	230	6.4
	9	11	5	5	25	2.3
	13	2760	2	745	10216	3.7
	14	1192	2	322	4414	3.7
	15	7718	2	2085	28569	3.7
	16	2815	2	760	10420	3.7
	17	350	2	95	1297	3.7
	18	3355	2	906	12421	3.7
	19	35	2	10	131	3.7
	20	215	2	58	797	3.7
Control	Human	50	5	22	115	2.3
	Mouse	17	4	7	43	2.5

Received 24 April 2013/1 July 2013; accepted 17 July 2013

Published as Immediate Publication 17 July 2013, doi 10.1042/BSR20130044

[1] To whom correspondence should be addressed (email mark.abbott@astrazeneca.com).

© 2013 The Author(s) This is an Open Access article distributed under the terms of the Creative Commons Attribution Licence (CC-BY) (http://creativecommons.org/licenses/by/3.0/) which permits unrestricted use, distribution and reproduction in any medium, provided the original work is properly cited.
